# Interaction between Occupational and Non-Occupational Arsenic Exposure and Tobacco Smoke on Lung Cancerogenesis: A Systematic Review

**DOI:** 10.3390/ijerph20054167

**Published:** 2023-02-25

**Authors:** Giuseppina Folesani, Maricla Galetti, Pier Giorgio Petronini, Paola Mozzoni, Silvia La Monica, Delia Cavallo, Massimo Corradi

**Affiliations:** 1 Department of Occupational and Environmental Medicine, Epidemiology and Hygiene, INAIL-Italian Workers’ Compensation Authority, Via Fontana Candida 1, Monte Porzio Catone, 00078 Rome, Italy; 2 Department of Medicine and Surgery, University of Parma, Viale Gramsci 14, 43126 Parma, Italy; 3 Center of Excellence for Toxicological Research (CERT), University of Parma, Viale Gramsci 14, 43126 Parma, Italy

**Keywords:** lung cancer, arsenic exposure, tobacco smoke, synergism, sub-multiplicative interaction, additive interaction

## Abstract

Although a higher lung cancer risk has been already associated with arsenic exposure, the contribution of arsenic and its compounds to the carcinogenic effects of other agents, such as tobacco smoke, is not well characterized. This systematic review examined the relationship between occupational and non-occupational arsenic exposure and tobacco smoking on lung cancer risk using papers published from 2010 to 2022. Two databases, PUBMED and Scifinder, were used for the searches. Among the sixteen human studies included, four were about occupational exposure, and the others were about arsenic in drinking water. Furthermore, only three case-control studies and two cohort studies evaluated an additive or multiplicative interaction. The interaction between arsenic exposure and tobacco smoke seems to be negligible at low arsenic concentrations (<100 μg/L), while there is a synergistic effect at higher concentrations. Finally, it is not yet possible to assess whether a linear no-threshold (LNT) model for lung cancer risk can be applied to the co-exposure to arsenic and tobacco smoke. Although the methodological quality of the included studies is good, these findings suggest that rigorous and accurate prospective studies on this topic are highly needed.

## 1. Introduction

Arsenic (As) is an element widely distributed throughout the environment in the air, land and water and is highly toxic in its inorganic form [1]. As reported by the WHO (World Health Organization) in 2003, an estimated 140 million people worldwide have been exposed to high levels of inorganic arsenic through the consumption of contaminated water, use of the same water for food preparation and irrigation and intake of relatively contaminated food [2]. Further, its use in consumer products was banned in 2004 in the US as well as in the EU [3]. The exposure to high levels of inorganic arsenic was also due to occupational exposure and tobacco smoking [2].

The International Agency for Research on Cancer (IARC) classified arsenic as a lung carcinogenic agent with sufficient evidence in humans [4]. Recently, the European Directive 2019/983 identified 0.01 mg/m^3^ as the occupational exposure limit value for arsenic acid, emphasizing that arsenic acid, its salts and inorganic arsenic compounds are carcinogens (category 1A) [5]. The National Institute for Occupational Safety and Health (NIOSH) established a recommended exposure limit (REL) for arsine of 0.002 mg As/m^3^ [6], the American Conference of Governmental Industrial Hygienists (ACGIH) defined the Threshold Limit Values (TLV-TWA) as 0.01 mg/m^3^ for arsenic and inorganic compounds and 0.005 ppm for arsine [6], while, in 2006, the US Environmental Protection Agency (EPA) set the maximum permitted concentration of arsenic in drinking water at 10 μg/L [7]. Long-term exposure to inorganic arsenic can lead to chronic arsenic poisoning, such as pigmentation changes and skin lesions (hyperkeratosis) and, in some cases, bladder and lung cancer [8,9]. However, as the symptoms and signs caused by long-term high exposure to inorganic arsenic differ between individuals, population groups and geographic areas, there is no universal definition of arsenic-related illness, which complicates the assessment of the health burden of arsenic.

Most of the papers in the scientific literature have focused on characterizing the risk associated with the oral intake of arsenic, considering arsenic in drinking water as the main source of exposure [10], while less attention has been paid to inhalation exposures, although the risk of developing lung cancer among miners of various ores and workers in metal smelters and refineries has increased [11,12]. Moreover, no method is available to distinguish cancer cases caused by arsenic exposure from those induced by other factors and, consequently, there is no reliable estimate of the extent of the problem worldwide.

Arsenic metabolism probably plays an important role in its carcinogenicity [13,14]. In vivo, this compound undergoes several complex metabolic conversions, but it is mainly metabolized by methylation into trivalent and pentavalent forms to enhance urinary excretion. Increased cancer risk has been associated with reduced methylation capacity, especially in the presence of high concentrations of arsenic when it could not be detoxified efficiently due to overwhelmed methylation capability [15]. Moreover, although methylation of arsenic has been considered a part of a detoxification pathway for many years, recently it has been proposed that intermediary metabolites are also reactive and toxic [16]. Therefore, it is possible to assume the presence of a balance between detoxification and carcinogenicity processes. Finally, arsenic could also enhance the carcinogenic effects of other carcinogenic agents, including tobacco smoke [17]. It has been reported that tobacco smoke and second-hand tobacco smoke, which IARC has classified as carcinogenic compounds for lung tissues [18,19], may be more hazardous for people co-exposed simultaneously to arsenic (inhaled or ingested). Thus, sometimes this association has resulted in a significantly higher proportion of subjects with lung cancer disease [20,21], even though further exploration of the type of interaction between arsenic and tobacco smoking is necessary for appropriate risk assessment and health surveillance.

Although data indicating a higher lung cancer risk has already been reported for arsenic exposure [22], the aim of this paper was to evaluate the latest quantitative evidence on the interaction between occupational and non-occupational exposures to arsenic and tobacco smoke on lung cancer risk using a systematic review of papers published in the last ten years. Simultaneously, we tried to focus on some unresolved issues: the presence or absence of a synergistic/additive interaction at both low and high arsenic exposure, the existence of a linear dose–response relationship (the linear no-threshold model, LNT) and the definition of arsenic, in interaction with smoking, as a carcinogen with or without a threshold dose value.

Being able to answer these questions means being able to better understand the mode of action (MOA) of arsenic exposure in lung carcinogenesis and, as already reported for radon, improve risk assessment and public health decision-making [23,24].

## 2. Materials and Methods

The “Items for Systematic Reviews and Meta-Analysis (PRISMA) guidelines”, as reported by Page and Moher [25,26], were used for this systematic review. The PRISMA review protocol was not registered.

### 2.1. Identification of Studies

A search of electronic literature bases (PubMed [*n* = 32] and Scifinder [*n* = 44]) was conducted in October 2022 using the keywords [arsenic and lung cancer and occupation* and non occupation* and smok*]. The bibliographic search was limited to those papers published from 2010 to 2022 and further relevant studies were added from the bibliography of the selected articles. We did not use gray literature.

Articles identified using the two bibliographic databases were checked for duplications, which were then removed from the reference list. The remaining records were reviewed for eligibility in a two-step process, conducted independently by two authors.

### 2.2. Inclusion Criteria

Human studies published in peer-reviewed journals reporting lung cancer and the interaction between occupational and non-occupational exposure to arsenic and smoking habits were included. All sex and age groups were considered. Prospective cohort, retrospective cohort, cross-sectional and case-control studies were included as eligible studies. Reviews were screened to retrieve relevant primary data. We considered lung cancer or lung cancer death as the primary outcome and selected only those studies that reported their estimated effect values: Odds Ratio (OR), Relative Risk (RR), Excess Relative Risk (ERR), Hazard Ratio (HR), number of deaths for lung cancer or urinary arsenic concentration as the measure for the assessment of exposure.

### 2.3. Exclusion Criteria

Papers that did not meet the inclusion criteria were excluded, which included in vitro studies, animal studies, book chapters, systematic reviews with and without meta-analyses, case reports and case series. Studies not published in English were not considered.

### 2.4. Data Extraction

Two screenings were performed: the first was on the basis of the title and abstract, and the second was on the full text availability. Disagreements between reviewers were resolved by discussion at both stages. A standardized data extraction spreadsheet was used for data extraction of the papers, which was completed by one author under the supervision of a second author.

The following data were extracted from each study included in the systematic review: authors’ first name, year of publication, study design (prospective cohort study, retrospective cohort study, population-based case-control study, cross sectional study), total number of subjects and gender (number of controls and cases and men and women), country, age, exposed study period, type of exposure, type of data collection, definition of smoking status, estimate effect type (ERR, HR, OR, RR, lung cancer death, geometric mean of urine arsenic) and estimate effect values, type of interaction (synergistic, additive and not reported) and NOS (Newcastle–Ottawa Assessment Scale) values. The characteristics of the included studies were reported for those performing a descriptive analysis. We recorded unadjusted and adjusted outcomes.

### 2.5. Quality Assessment

We used the Newcastle–Ottawa Assessment Scale (NOS) [27] for assessing the quality of the included studies. As reported by Montazeri et al., the scale has a minimum score of zero and a maximum score of nine. Studies scoring seven or more (corresponding to 78% of the maximum score) were considered as having a low risk of bias (‘good’ quality); studies scoring four-six were considered at a modest risk of bias (‘fair’ quality); and studies with scoring < three were considered at a substantial risk of bias (‘poor’ quality) [28]. An exclusion criterion was not represented by the quality level of the study. The various points of disagreement between the reviewers were resolved through discussion.

## 3. Results

### 3.1. Study Selection

**Figure 1:**
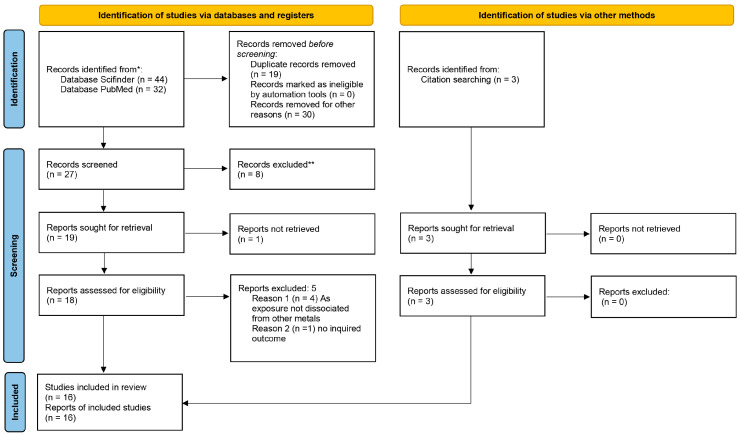
Prisma flow chart of the included studies [25]. * The bibliographic search was limited to those papers published from 2010 to 2022. ** The exclusion was done through discussion between the reviewers.

As shown in Figure 1, according to the primary publication research protocol described in the Methods section, sixteen studies were included for quantitative synthesis of the role of arsenic and smoking habits in lung carcinogenesis.

Specifically, 76 eligible articles were enrolled: 32 from PubMed and 44 from Scifinder. Nineteen duplicated papers and thirty unrelated articles were excluded, leaving twenty-seven full-text articles assessed for eligibility.

Further, another fourteen articles were removed: one review, one congress abstract, eight due to the topic, two due to incomplete information about the interaction between arsenic exposure and smoke, one due to language and one that was not available.

Ultimately, of the 19 studies selected for the qualitative synthesis, we obtained 16 articles suitable for quantitative synthesis (including 3 from the reference list of the selected articles) (Figure 1).

### 3.2. Characteristics of the Included Studies

Sixteen articles were identified for the arsenic–smoking interaction in lung cancer risk and full-text articles were found to be eligible. The main characteristics of these 16 studies are listed in Table 1. Study enrolment periods ranged from a minimum of two years [29] to a maximum of twenty-seven years, including follow-up periods [30].

Of the selected studies, fifteen studies were retrospective and only one was a prospective study. The retrospective studies included three cohort studies [30,31,32], two multicenter case-control studies [33,34], seven case-control studies [29,35,36,37,38,39,40], one cross-sectional study [41], one population-based study [42] and one review of epidemiological data [43]. The latter included an analysis of new data from the National Health and Nutrition Examination Survey (NHANES), so it was evaluated for eligibility in this systematic review.

**Table 1:** Characteristics of the included studies.

Author (Year)	Study Design	Subjects	Country	Age (Years)	Exposed Study Period	Data Collection	NOS *
Su Z. et al., 2022 [30]	Cohort	M/W (1215/405)	Taiwan	≥40	1992–2019	Baseline questionnaire and face to face interview	9
Fan Y. et al., 2016 [44]	Prospective occupational-based cohort	M/W (8696/599)	China	40–59; 60–69; >70	1992–2001	Personal interview	5
Steinmaus C.M. et al., 2015 [29]	Case-control	M/W (637/298) Cases: 301 Controls: 634	Chile	Case: 65.9 ± 10.2 Control: 66.3 ± 11.3	2007–2009	Standardized study questionnaire and government agencies, research studies	6
D’Ippoliti D. et al., 2015 [42]	Population-based	M/W (82,169/83,440)	Italy	≤35; 36–64; >65	1990–2010	Population registries	4
Steinmaus C.M. et al., 2014 [35]	Case-control	M/W (257/123) Cases: 92 Controls: 288	Chile	25–65	2007–2010	Face to face interview and questionnaire	8
Steinmaus C.M. et al., 2013 [36]	Case-control	M/W (601/271) Cases: 306 Controls: 640	Chile	30–39; 40–49; 50–59; 60–69; 70+	1958–1970	Standardized questionnaire	8
Hsu L. et al., 2013 [31]	Cohort	M/W (1231/1216)	Taiwan	30–49; 50–59; 60–69; ≥70	1985–1989	Interviewed at homewith a structured questionnaire	5
Ferreccio C. et al., 2013 [37]	Case-control	M/W (646/300) Cases: 306 Controls: 640	Chile	30–39; 40–49; 50–59; 60–69; 70+	2007–2010	Standardized study questionnaire and government agencies, research studies	6
Dauphiné D.C. et al., 2013 [38]	Case-control	M/W (237/318) Cases: 196 Controls: 359	Nevada and California (USA)	Case: mean 70.2 ± 10 Control: mean 69.0 ± 8.6	2002–2005	Standardized study questionnaire by telephone	8
Chen C. et al., 2010 [32]	Cohort	M/W (3481/3407)	Taiwan	≤50; 50–54.9; 55–59.9; ≥60	1991–2002	Home interview with a questionnaire	7
Paul S. et al., 2013 [41]	Cross-sectional	2005–2006 88 M, unexposed 83 W, unexposed 94 M, exposed 95, W exposed 2010–2011 87 M, unexposed 83 W, unexposed 85 M, exposed 91 W, exposed	India	2005–2006 34.76 ± 9.52 unexposed 34.62 ± 12.93 exposed 2010–2011 39.05 ± 9.08 unexposed 38.65 ± 13.09 exposed	2005–2010	Expert physician screening	4
Marano K.M. et al., 2012 [43]	Review of epidemiological data	Cigarette smokers, *n* = 991 Smokeless tobacco (SLT) consumers, *n* = 90 Non-consumers of tobacco *n* = 3385	USA	≥20	2003–2008	Database (NHANES: National Health and Nutrition Examination Survey)	2
Wadhwa S.K. et al., 2011 [39]	Case–control	Exposed subjects 98 M, referents without cancer 52 M, with lung cancer Non-exposed subjects 95 M, referents without cancer 55 M, with lung cancer	Pakistan	median 47 (range 35–65)	2007–2009	Face to face interview and medical database	4
Olsson A.C. et al., 2011 [33]	Multicenter case-control	M/W (4054/1260) Cases: 2624 Controls:2690 M/W exposed (91/5) Exposed cases: 60 Exposed controls:36	Czech Republic, Hungary, Poland, Romania, Russia and Slovakia	<45; 45 to 49; 50 to 54; 55 to 59; 60 to 64; 65 to 69; 70 to 74; 75+	1998–2002	Face to face interview and questionnaire	7
’t Mannetje A. et al., 2014 [34]	Multicenter case-control	M/W (4492/1464) Cases: 2852 Controls: 3104 Exposed cases: 70 Exposed controls:43	Romania, Hungary, Poland, Russia, Slovakia, Czech Republic and UK	25–45; 45–55; 55–65; 65 or older	1998–2003	Face to face interview and questionnaire	7
Melak D. et al., 2014 [40]	Case-control	Cases: 94 Controls: 347	Chile	>25	2007–2010	Face to face interview, questionnaire and medical database	8

* NOS: the Newcastle-Ottawa Assessment Scale was used to assess the quality of nonrandomised studies with its design, content and ease of use, directed to the task of incorporating the quality assessments in the interpretation of meta-analytic results.

Of the three selected cohort studies [30,31,32], all conducted in Taiwan, Su’s study [30] was on occupational exposure, while the others two were on non-occupational exposure to arsenic in drinking water. Su et al. [30] recruited 1215 men and 405 women aged 40 years or older, studying an exposure period of approximately 30 years. Hsu et al. [31] and Chen et al. [32] jointly recruited a total of 4712 men and 4623 women for an exposure period of 4 and 11 years, respectively. From Chen’s work [32], data on arsenic concentrations were reported for 6888 subjects, of which 178 cases had been diagnosed with lung cancer (with a lung cancer incidence of 2.24 cases per 1000 person-years).

The first multicenter case-control study [33] was conducted in the Czech Republic, Hungary, Poland, Romania, Russia and Slovakia, and analyzed 2624 cases and 2690 controls, after having recruited a total of 4054 males and 1260 women. This study enrolled subjects with exposure to almost 70 different occupational agents. The second multicenter [34] study was conducted in the same countries listed above and additionally in the U.K.; it recruited 4492 cases and 1464 controls.

The seven case-control studies, on the other hand, included a smaller number of cases, ranging from a minimum of 92 [35] to a maximum of 306 [36], and were also conducted in three countries: Chile, the USA and Pakistan [29,35,36,37,38,39,40]. Study enrolment periods ranged from a minimum of two [29,39] to a maximum of twelve years [36]. All thepapers did not report the type of occupational exposure but evaluated exposure to drinking water with a high arsenic content as a possible confounding factor interfering with occupational risks.

The cross-sectional study [41] and the population-based study [42], conducted in India and Italy, respectively, examined non-occupational arsenic drinking water exposure. In particular, the cross-sectional study assessed effects during the period from 2005 to 2010, recruiting approximately 90 males and 90 females, exposed and unexposed [41], while the population-based study assessed effects over a 20-year exposure period, recruiting 82,169 males and 83,440 females [42].

As previously reported, the review of epidemiological data from the of National Health and Nutrition Examination databases in the US was included, as it evaluated new occupational and non-occupational exposure data over a five-year period [43].

Finally, the only prospective study included in Table 1 was a cohort study, recruiting 9295 tin miners (93.6% men) working in China [44]. Therefore, only occupational exposure to arsenic was evaluated in this article.

### 3.3. Risk of Bias in Included Studies

The study quality of the enrolled articles was mainly moderate to high, with a low risk of bias, as shown by the NOS value reported in Table 1 and Table S1.

In particular, eight out of the sixteen included studies were evaluated as high-quality research, scoring 7–9, while two papers were evaluated as moderate-quality research, scoring 6 (Table 1 and Table S1) and another six were reported as low-quality research.

**Figure 2:**
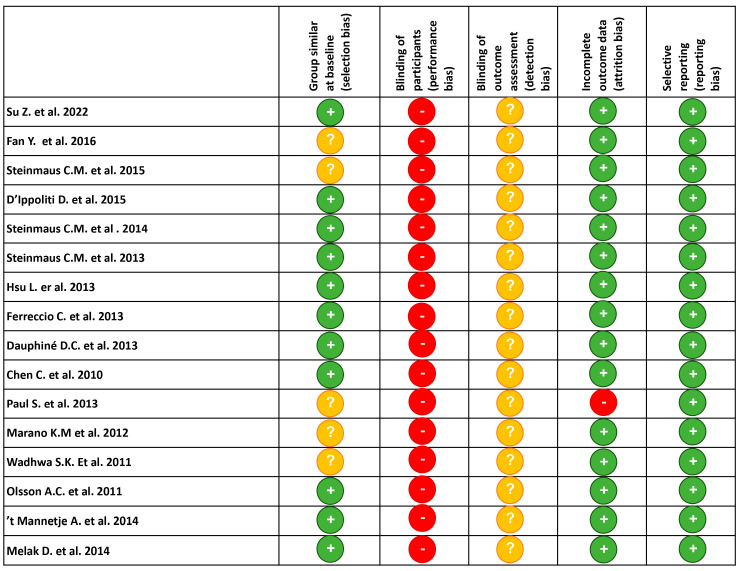
Assessment risk of bias [29,30,31,32,33,34,35,36,37,38,39,40,41,42,43,44]. Green circles with “+” sign indicate low risk, yellow circles with “?” sign indicate unclear risk, red circles with “-“ sign indicate high risk.

All studies were susceptible to mild cohort selection bias, due to their retrospective study design, with the only exception being the prospective occupational-based cohort [44]. In addition, because the assessment of outcomes was performed using a medical record review, we regarded it as susceptible to mild outcome bias. The selection bias is reported in Figure 2 for every study included in Table 1.

In many of the selected studies, exposure to arsenic mainly concerned subjects exposed to naturally occurring arsenic in drinking water [29,31,32,35,36,38,39,40,41,42], whereas only four studies showed true occupational exposure to arsenic [30,33,34,44] (Table 2). Furthermore, the type of occupational exposure has not always been characterized; in fact, Ferreccio et al. [37] studied workers exposed to copper and other minerals and arsenic exposure derived mainly from drinking water, while Marano et al. [43] reported occupational and non-occupational exposure, based only on a review of epidemiological data.

Moreover, although most studies recruited mainly men, one paper investigated the consequences of arsenic exposure in utero from arsenic-contaminated water [45]. However, this study was not included in our systematic review because the authors did not evaluate the effect of smoking on lung cancer disease.

All studies correctly provided the data source and the methods used for their collection. Personal and medical information was mostly collected from medical records (performance bias), occupational histories from employment records and smoking habits from questionnaires and, for the D’Ippoliti study [42], from tobacco sales in the different municipalities. Therefore, their reliability may be affected by the recall bias resulting from the questionnaires used to collect retrospective data and the quality of the record documentations. Only in the paper of Fan et al. [44], where past lung diseases were reported as diagnosed by the physician, it was possible to minimize the recall bias to some extent. Moreover, although data collection could be complete and accurate, it must be emphasized that the methods used to identify and assess occupational exposures may also be a source of bias especially for the D’Ippoliti’s study [42] [Figure 2].

### 3.4. Arsenic–Smoking Interaction

The characteristics and the results of the sixteen selected studies evaluating arsenic-smoking statistical interaction are presented in Table 1 and Table 2.

Out of the sixteen studies, eleven of them did not reveal any interaction when they were assessed [29,31,33,34,35,38,40,41,42,43,44], while three case-control studies [36,37,39] and two cohort studies [30,32] evaluated an additive or multiplicative interaction.

Among the types of study designs included in our systematic review, there is only one paper with a prospective cohort design, which studied a large cohort of Chinese tin miners and examined interactions between occupational arsenic and previous lung disease on lung cancer risk [44]. The estimated effect for miners exposed to arsenic and other carcinogenic compounds was reported as HR for never smokers (HR = 1), former smokers (HR = 1.31 (95% CI: 0.77–2.24)) and current smokers (HR = 1.53 (95% CI: 0.95–2.44)) (Table 2). However, the adjustment for smoking was not found to be statistically significant. Nevertheless, a multiplicative or additive interaction between previous lung diseases, such as asthma (negative interaction), and arsenic exposure has been reported. Furthermore, the authors provided strong evidence that arsenic exposure is associated with lung cancer risk; in fact, HR increased as the arsenic concentrations increased.

Regarding the other cohort studies, Su et al. [30] evaluated the effects of arsenic exposure on tin miners working for a company in Taiwan. The authors reported the ERR values for lung cancer at different arsenic exposures (0.0033 (95% CI: 0.0014–0.0045) and 0.0056 (95% CI: 0.0035–0.0073) for arsenic concentrations <3 mg/m^3^ and ≥3 mg/m^3^, respectively) and defined the type of interaction between arsenic exposure and tobacco smoking (Table 2). In particular, the results of the sensitivity analysis indicated the presence of a sub-multiplicative interaction between these two agents, with a higher risk of lung cancer for short-term exposures to high arsenic concentrations than for long-term exposures to low concentrations.

Hsu et al. [31] and Chen et al. [32] also described two cohort studies, analyzing the effects of arsenic exposure in drinking water on a large population in Taiwan. Hsu et al. [31] reported the lung cancer risk for smokers and non-smokers by estimating the type of effect as HR for hyperkeratosis and/or skin cancer, while Chen et al. [32] referred to the effect estimated as RR at arsenic concentrations ≥ 100 µg/L (1.32 (95% CI: 0.64–2.74) for never smokers, 5.30 (95% CI: 2.19–12.8) for smokers < 25 pack years and 8.17 (95% CI: 3.74–17.9) for smokers ≥ 25 pack years) (Table 2). While the type of interaction between arsenic exposure and smoking was not reported in Hsu’s paper, in Chen’s study, it was defined as synergistic for squamous and small-cell lung cancer.

When we evaluated the risk of lung cancer attributable to the co-exposure to arsenic and smoke, in the nine selected case-control studies, we observed two different types of arsenic exposure [29,33,34,35,36,37,38,39,40]: seven studies reported on exposure in drinking water [29,35,36,37,38,39,40], while the last two papers dealt with occupational exposure [33,34].

Steinmaus et al. [29] showed that for the same concentrations of arsenic in drinking water, the OR values were higher for subjects with high BMI (body mass index) than for those with low BMI (Table 2). Although the data confirmed a synergistic relationship between high BMI and arsenic exposure in association with lung cancer, adjustments for smoking had minimal impact.

In another case-control study conducted in Chile, the same author [35] focused on the association between low arsenic exposure in drinking water (<100 µg/L) and lung cancer risk. At arsenic concentrations in drinking water greater than 59.9 μg/L, the OR crude value was 1.90 (90% CI: 1.16–3.13) and 2.01 (95% CI: 1.14–3.52) when adjusted for age, sex and smoking behavior (Table 2). However, the type of interaction, additive or synergic, between arsenic exposure and smoking was not discussed.

Another paper by Steinmaus et al. [36], also conducted in Chile, showed clear evidence of dose–response relationship between arsenic exposure and ORs for lung cancer, adjusted for smoking. In particular, lung cancer ORs, calculated before 1971 and adjusted for quartiles of average arsenic concentrations in water, ranged < 11; 11–90; 91–135; >335 µg/L, were 1.00, 1.27 (95% CI: 0.81–1.98), 2.00 (95% CI: 1.24–3.24) and 4.32 (95% CI: 2.60–7.17), respectively. Note that Steinmaus et al. reported higher ORs only when the exposures were prior to 1971 because, in that year, the very high exposures in the city of Antofagasta ended. Finally, although this study only showed smoke-adjusted values and not raw values, it defined that arsenic exposure and smoking act synergistically in inducing lung cancer.

Similar results were obtained by Dauphiné et al. [38] in a case-control study conducted in Nevada and California (USA) investigating arsenic exposure in drinking water. Indeed, at arsenic concentrations ≥85 µg/L, the OR value was 1.39 (95% CI: 0.55–3.53) for all subjects and 1.61 (95% CI: 0.59–4.38) for smokers (Table 2). However, the reported OR values were not statistically significant compared to those presented by Steinmaus et al. [36] and, at a concentration near 100 µg/L, no association with adverse health risks was found.

Another case-control study analyzing arsenic exposure in drinking water is reported by Ferreccio et al. [37]. This study analyzed data on people exposed to both arsenic and other known or suspected carcinogens, including tobacco smoke and compounds such as wood dust, silica and asbestos. In subjects who smoked more than 10 cigarettes/day, the OR values for lung cancer risk increased as the concentration of arsenic in drinking water increased, reaching a value of 20.80 (95% CI: 9.03–47.91) at concentration > 260 µg/L. Evidence of more than additive effects on lung cancer risk was found in people co-exposed to arsenic and smoking (Table 2).

Wadhwa et al. [39], analyzing only smoking subjects, reported an OR value of 3.05 (95% CI: 1.26–7.36) for lung cancer mortality among patients exposed to arsenic compared to subjects with lung cancer not-exposed to arsenic. The authors also reported an increased risk of cancer due to a synergistic association between arsenic ingestion and tobacco smoke (Table 2).

Finally, Melak et al. [40] evaluated the effects of arsenic exposure in drinking water, measuring inorganic arsenic and, for the first time, its metabolites such as monomethylarsonous acid (MMA3), monomethylarsonic acid (MMA5) and dimethylarsenic acid (DMA5) in urine. The author reported the effect estimated as OR at water arsenic concentrations < 200 µg/L and ≥200 µg/L, further differentiating between the proportion of inorganic arsenic excreted in urine reported as MMA (MMA3 plus MMA5). The OR crude and adjusted values were reported in Table 2, even though there was not a significant difference between these OR values.

Unlike previous studies, the last two case-control studies reported lung cancer risk for occupational arsenic exposure considering the interaction with smoking [33,34].

Olsson’s multicenter case-control study [33] reported an OR for men of 1.92 (95% CI: 1.15–3.20) and for women of 1.05 (95% CI: 0.11–9.89), adjusted for smoking. Although it is indicated that tobacco smoking modifies the effect of occupational exposure to arsenic, unadjusted values were not reported. Finally, ’t Mannetje et al. [34], the last multicenter case-control study, reported on the association between occupational exposure to metals (including chromium, cadmium, nickel and arsenic compounds) and lung cancer risk in the agriculture and chemical industries, adjusting for confounding factors such as smoking habits. Exposure to arsenic, which had a prevalence of about 1.4%, lower than the other metals, was associated with an increased risk of lung cancer (OR = 1.65 (95% CI: 1.05–2.58)). However, no additive or synergistic interactions have been reported (Table 2).

Only one cross-sectional study appears in this systematic review [41], in which, in the arsenic-exposed population, there were three deaths from lung cancer, while in the non-exposed population, there was only one individual who died of natural causes. Once again, however, no additive or synergistic interactions were reported.

Finally, we included in this systematic review a population-based study [42] and a review of epidemiological data [43]. The former, D’Ippoliti et al. [42] reported for low arsenic concentration (10–20 μg/L) a higher HR ratio for death from lung cancer than for death from natural cases in both female and male populations (Table 2). However, the authors did not report a dose–response relationship between arsenic exposure and lung cancer risk. Unusually, in contrast to other studies, the hazard ratio was adjusted for smoking sales from different municipalities.

The second, Marano et al. [43] found a higher concentration of arsenic in the urine of subjects with lung cancer risk compared to the values of smokers and non-consumers of tobacco without lung cancer. The difference was statistically significant, but no additive or synergistic interaction was reported (Table 2).

### 3.5. Outcome Measures

The outcome was the diagnosis of lung cancer or lung cancer death and, for only one study, the urinary arsenic concentration [43]. The effect estimated as OR, HR, RR and ERR was available for most studies. For two studies, the estimated effect was the number of lung cancer deaths [41] and the geometric mean of arsenic in urine [43], respectively (Table 1 and Table 2).

## 4. Discussion

### 4.1. Summary of Findings

This systematic review comprises four cohort studies, including one prospective study, and nine case-control studies, of which two multicentric studies, one cross-sectional study, one population-based study and a review of epidemiological data. The main finding can be summarized as follows:

- Four of the selected studies dealt with occupational exposure to arsenic [30,33,34,44]; the others concerned drinking water or food containing arsenic.
- Five studies have identified a synergism between arsenic dose and cigarette smoking in the induction of lung carcinoma [30,32,36,37,39]. In particular, Su et al. [30] found a sub-multiplicative interaction and Ferreccio et al. [37] referred to a “greater than additive effects”, while the others defined a synergistic interaction.
- Synergism with smoking significantly changed the lung cancer risk when exposed to higher versus lower concentrations of arsenic.
- Some studies did not have complete quantitative characterization of exposure [34]. Conversely, Ferreccio et al. [37] asserted that “his study took place in an area with a history of high concentrations of arsenic in drinking water and good data on past exposure”. Moreover, detailed tobacco consumption was sometimes missing; for example, D’ippoliti et al. [42] reported indirect data on cigarette sales in different municipalities as an indicator of smoking status.

### 4.2. Strengths and Limitations of This Systematic Review

Although this systematic review was not registered in PROSPERO, several strengths are present, such as an appropriate and robust study methodology and a clear scope, which reflects on the important aspect of the type of interaction between arsenic and smoke exposure. Moreover, we predefined inclusion/exclusion criteria for subjects/workers population, interventions, comparators, outcomes and study design. Similarly, the literature search was conducted with a pre-set search strategy, in two different databases (PUBMED and Scifinder). We tried to reduce the risk of publication bias by also avoiding any language restriction, although in a Chinese study, it was not possible to evaluate the published data. In addition, a manual search from reference lists of the selected articles was also added.

Two reviewers carried out the study selection independently and disagreements were resolved through discussion. The characteristics of each included study were provided in adequate detail.

This review also has some limitations such as the small number of included studies with occupational exposure [30,33,34,43,44] and the high abundance of retrospective studies, which could introduce information bias.

Finally, the definition of smoking status was not always well characterized in all studies due, for example, to the fact that in one study, tobacco consumption was assessed based on cigarette sales in municipalities [42].

### 4.3. Interaction between Arsenic and Tobacco Smoke and Determination of Estimate Effect Values

The literature on arsenic in drinking water and lung cancer has focused mainly on areas contaminated with high arsenic concentrations [46] and, only in the last years, on low arsenic exposures [47,48]; moreover, the type of interaction with cigarette smoke has not always been evaluated. In addition, less attention has been focused on occupational exposure to arsenic, although the risk of developing lung cancer among miners and workers in metallurgical and petrochemical industries has increased [49,50].

We reviewed the literature on the interaction between occupational and non-occupational exposures to arsenic and tobacco smoke on lung cancer risk over the past decade.

Among the papers dealing with occupational exposure to arsenic, Fan et al. [44] showed that tobacco smoking did not have significant effects on the HR values between never, former and current smokers in a large prospective cohort of China’s tin miners. However, this study provided evidence that arsenic exposure was associated with lung cancer risk, showing an increase in HR values as arsenic concentrations increased, at concentrations between 0 and 17,435 mg/m^3^ of cumulative arsenic exposure.

In contrast, Olsson et al. [33] provided statistically significant OR values for lung cancer risk adjusted for smoking, even though the results were significant only for men and not for women. Furthermore, a stronger effect of occupational exposures to arsenic has been reported among current smokers, suggesting that smoking may modify the effect of occupational exposures. The cut-off levels for arsenic exposure indicated were a < 50% threshold limit value (TLV) for “low”, 50–150% TLV for “medium”, and >150% TLV for “high”.

Further confirmation came from ‘t Mannetje’s study [34], which reported statistically significant smoking-adjusted OR values for lung cancer risk. It should be noted that the OR values for exposure to dust and fumes/mist of arsenic, chromium, nickel and cadmium were adjusted not only for smoking but also for age, center, sex and exposure to other occupational agents, including the metals under study. The ORs were greater than one for all the metals under investigation, and the highest risk was observed for arsenic (OR = 1.65 (95% CI = 1.05–2.58)), thus underlining how the increased risk of lung cancer is associated with arsenic exposure. Moreover, for all metals, the adjustment for these agents led to an attenuation of the OR, whereas for arsenic, this adjustment did not affect the exposure–response relationship. The conclusions of this study have some limitations. Data were corrected for smoking habit using unconditional logistic regression, but the weight of this correction could not be assessed because raw ORs or ORs for smokers vs. non-smokers were not reported. In addition, cumulative exposure to arsenic and its compounds were classified as low, medium and high, but the arsenic concentration values were not reported.

However, none of the above-mentioned studies focused on the type of arsenic–smoke interaction. The only occupational study reporting this information was that of Su et al. [30], which pointed a sub-multiplicative interaction between arsenic and smoking in a cohort of tin miners, showing a higher ERR for cumulative arsenic exposure ≥ 3 mg/m^3^ compared to < 3 mg/m^3^. However, in this paper the mean cumulative arsenic exposure was 83.60 mg/m^3^, which is distant from the cut-off value of 3 mg/m^3^. Importantly, this paper, with its high NOS value, is the first study that quantitatively highlighted the important role of tobacco smoke as a co-factor for lung cancer at high arsenic concentrations. Indeed, although Olsson et al. stated that the effect of arsenic exposure was stronger in smokers, he did not report the arsenic concentrations to which workers were exposed.

Even though the interaction between occupational arsenic exposure and smoking has been documented before [17,51], it is very important to confirm that smoking may be a confounder or effect modifier in occupational arsenic exposure. Indeed, failure to evaluate this interaction may lead to a significant degree of uncertainty in the evaluation of the dose–response relationship between arsenic exposure and lung cancer or lung cancer death. Moreover, when assessing occupational exposure to arsenic, it is also possible to have a co-exposure with other lung carcinogens such as radon and heavy metals. For instance, Fan et al. [44] adjusted arsenic exposure for lung cancer risk with radon exposure, showing that the confounding effect of radon may not be fully excluded. Furthermore, t’Mannetje et al. [34] adjusted arsenic exposure for lung cancer risk with co-exposure to chromium, cadmium and nickel. The authors evidenced that arsenic exposure had the strongest risk, but chromium had the most important contribution due to its high prevalence. Finally, in most of these studies, information about the use of workers’ protective respirators was reported.

While occupational arsenic exposure was considered by few articles included in this systematic review, more studies have evaluated the effect of ingested arsenic, especially through drinking water.

The case-control study investigated by Ferreccio et al. [37] dealt with carcinogenic compounds and concurrent exposure to arsenic in drinking water, showing a 10-fold higher risk of lung cancer in co-exposed people than in unexposed people. Again, as in Su’s paper [30], the synergistic effect between arsenic and smoke was particularly evident at high arsenic concentrations. Interestingly, in Ferreccio’s study [37], synergy indices were substantially higher than 1.0, suggesting that the combined effects of the multiple carcinogens analyzed are greater than the additive effects (Rothman Synergy Index = 4.0 (95% CI: 1.7–9.4), also in people co-exposed to arsenic and second-hand tobacco smoke. Moreover, these indices tended to be elevated for agents already known or suspected to cause lung cancer (e.g., arsenic and asbestos), but not for agents not linked to lung cancer (e.g., solvents). Even though some data were already known, and a synergistic relationship between arsenic and smoking in lung cancer risk has been previously shown [20,52], the novelty of this study is the analysis of the combined effect of arsenic with second-hand tobacco smoke.

Concerning exposure to arsenic in drinking water, eleven further papers were included in this systematic review [29,31,32,35,36,37,38,39,40,41,42]. Most of them were conducted by the same group of researchers, who performed case-control studies of lung cancer in areas of Chile. Steinmaus et al. [36] reported a dose–response relationship between arsenic concentration and lung cancer and its synergy with smoking behavior (stated, but not mathematically demonstrated), while additional adjustments for exposure to second-hand smoke had only small effects on the ORs. Moreover, for the first time, he found clear evidence of an elevated arsenic-related cancer risk in people exposed also to low arsenic concentration, almost after 40 years of cessation of high exposure to drinking water [36], as evidenced by the 4-fold increases in OR values. Thus, similar to mesothelioma caused by asbestos [53], the high risk of cancer linked to arsenic in drinking water continues for decades after exposure has ceased [54], while the risk of tobacco-related cancers is known to decrease within a few years after smoking cessation, approaching the risks of non-smokers within few decades [55]. Probably, the very long latency period between exposure and cancer disease appeared to be one the most common cause of arsenic-related death, even though the mechanism by which arsenic may increase long-term cancer risk is still unknown. Therefore, it is very important to eliminate exposures as soon as possible, and public health interventions are needed for many years after exposures cease.

Similarly, Dauphiné et al. [38] reported on another case-control study that examined lung cancer risk associated with arsenic in a population exposed to low to moderate arsenic levels in drinking water (between 50 and 100 µg/L). Although the odds ratios were slightly higher in the analyses of smokers, the statistical power was not sufficiently adequate to assess synergy due to the small number of non-smokers. Therefore, it was not possible to suggest a marked increased risk of lung cancer in people exposed to arsenic in drinking water at concentrations close to 100 µg/L. Furthermore, as previously reported, the authors again suggested an arsenic-related cancer latency of 40 years or more.

Instead, Steinmaus et al. [35] focused on lower long-term exposures and provided new evidence that arsenic concentrations in water below 100 µg/L were associated with a higher lung cancer risk, with greater odds ratios in younger adults and persons with early-life exposure. However, the smoking correction did not change the OR values and, therefore, this factor seemed negligible. In addition, the authors were not able to examine the risks for exposure at concentrations below the US standard (10 µg/L) because this would require very detailed information on confounding factors [56]. Similarly, the smoking correction did not change the OR values in Melak’s study [40], which evaluated high and low arsenic concentrations in drinking water. Even though he did not discuss the type of interaction between arsenic exposure and smoking, he considered the cut-off value of 200 µg/L to discriminate between low and high arsenic exposures. This work highlighted the need to plan new studies to determine the exact cut-off value (100, 200 or other value) to differentiate low or high exposure. Notably, for the first time, Melak et al. made speciation of methylated forms of arsenic by correlating them with exposure and risk of lung cancer.

Since susceptibility to arsenic varies widely from person to person, and risks may be even higher in certain susceptible sub-populations, Steinmaus et al. [29] also suggested the need to analyze the interaction between arsenic and some physical characteristics, such as body mass indices (BMI). Preliminary evidence showed that environment-related cancer risks may be significantly increased in people with high BMIs, even though in these subjects, the adjustments for smoking had little impact on the synergistic relationships between arsenic exposure and lung cancer.

With respect to the different histotypes of lung cancer, Chen et al. [32] observed a synergistic effect for the arsenic–smoking interaction in squamous and small carcinoma but not in adenocarcinoma. Furthermore, for lower exposure levels, he confirmed a dose–response relationship between lung cancer risk at increasing arsenic concentration (7-fold increased risk for heavy smokers at arsenic exposure level ≥ 100 µg/L respect to never smokers).

Another paper [39] found a synergism between arsenic and smoking at arsenic concentrations around 100 µg/L or higher (with a maximum of 150 µg/L), while D’Ippoliti et al. [42] and Hsu et al. [31] did not report the dose–response association. Although, Hsu referred to arsenic as a co-carcinogenic compound with other carcinogens, including tobacco smoke.

In conclusion, the smoke–arsenic interaction was negligible at low arsenic concentrations (<100 µg/L), while there was a synergistic effect at high exposures on lung cancer risk, as reported in four of the included studies [30,32,36,37] and in agreement with previously published papers. Nevertheless, the previously published papers discussed the synergism between arsenic exposure and smoke without distinguishing between low and high exposure [20,52].

In addition, the exact cut-off value of co-exposure to arsenic and smoking, which can induce lung cancer, will have to be precisely determined using further studies. Indeed, as reported by Lamm et al. for arsenic exposure alone [48], the cancer risk from ingesting high levels of arsenic in drinking water is evident, with a cut-off value higher than 200 µg/L, while the risk and the risk pattern at low levels of arsenic intake is uncertain. Frequently, the dose–response relationship at low levels is not represented by the LNT model but by alternative models (e.g., the linear-quadratic model) that can better explain the risk pattern.

## 5. Conclusions

The incidence of lung cancer risk in the workplaces is related to the type of carcinogenic compounds and their concentrations, but this could be influenced by the interaction of carcinogens with other carcinogenic compounds including tobacco smoke.

This systematic review provided evidence that the interaction between arsenic exposure and tobacco smoke seems to be negligible at low arsenic concentrations (<100 µg/L), while there is a synergistic effect at high concentrations of this compound. The exact cut-off value will have to be determined accurately using further studies, preferably cohort and prospective. Indeed, the reported value to date ranges between 100 µg/L and 200 µg/L.

Furthermore, it would be very interesting to define the role of arsenic as a co-carcinogen compound in the presence of other carcinogens not just tobacco smoke. Considering that tens of millions of people are exposed to arsenic worldwide, and that many of these people are likely to be co-exposed to at least one other known or suspected carcinogen, including tobacco smoke, primary prevention aiming to reduce environmental exposure to arsenic from drinking water, food and from workplaces should be the prioritized approach.

Finally, with the available data, it is not yet possible to assess whether arsenic exposure with co-exposure to tobacco smoke can be considered a threshold or not-threshold dose (applying LNT model) exposure for lung cancer risk, and further studies are necessary.

## Figures and Tables

**Table 2:** Quantitative analysis of the included studies.

Author (Year)	Type of Exposure	Smoking Status	Estimate Effect Type	Estimate Effect Value			Type of Interaction
Su Z. et al., 2022 [30]	Occupational: tin miners (Chinese Yunnan tin corporation)	Never smoker Current smoker	ERR	Mean cumulative arsenic exposure: 83.6 mg/m^3^			Sub-multiplicative
				Cumulative arseinc exposure < 3 mg/m^3^ *			
				0.0033 (95% CI: 0.0014–0.0045)			
				Cumulative arseinc exposure ≥ 3 mg/m^3^ *			
				0.0056 (95% CI: 0.0035–0.0073)			
				* Adjusted for smoking			
Fan Y. et al., 2016 [44]	Occupational: tin miners	Never smokers Former smoker Current smoker	HR	Cumulative arsenic exposure between 0 and 17,435 mg/m^3^			Not reported
				1.00	Never smokers *		
				1.31 (95% CI: 0.77–2.24)	Former smokers *		
				1.53 (95% CI: 0.95–2.44)	Current smokers *		
				* adjusted for all prior lung diseases and different carcinogenic compounds exposure			
Steinmaus C.M. et al., 2015 [29]	Non-occupational: arsenic drinking water	Never smokers Ever smokers Heavier smokers (smoking > 20 cigs/day)	OR	LOW BMI, Unadjusted			Not reported
				1.00	[<100 μg/L]		
				1.24 (95% CI: 0.84–1.82)	[100–800 μg/L]		
				2.47 (95% CI: 1.75–3.49)	[>800 μg/L]		
				LOW BMI, Adjusted *			
				1.00	[<100 μg/L]		
				1.17 (95% CI: 0.79–1.73)	[100–800 μg/L]		
				2.31 (95% CI: 1.63–3.29)	[>800 μg/L]		
				HIGH BMI, Unadjusted			
				1.00	[<100 μg/L]		
				1.52 (95% CI: 0.56–4.15)	[100–800 μg/L]		
				5.83 (95% CI: 1.73–19.64)	[>800 μg/L]		
				HIGH BMI, Adjusted *			
				1.00	[<100 μg/L]		
				1.48 (95% CI: 0.53–4.14)	[100–800 μg/L]		
				6.98 (95% CI: 1.84–26.56)	[>800 μg/L]		
				* Adjusted for age, sex and smoking			
D’Ippoliti D. et al., 2015 [42]	Non-occupational: arsenic drinking water	Not directly determined (used tobacco sales from municipal level assumption)	HR	Arsenic (10–20 μg/L) *			Not reported (dose response relationship)
				1.27 (95% CI: 1.18–1.38)	Natural causes, males		
				1.47 (95% CI: 1.17–1.86)	Tracheas, bronchus and lung, males		
				1.14 (95% CI: 1.05–1.24)	Natural causes, females		
				1.80 (95% CI: 1.23–2.66)	Tracheas, bronchus and lung, females		
				Cumulative Arsenic dose (CAI; 204.9–804.0 μg) *			
				1.59 (95% CI: 1.45–1.74)	Natural causes, males		
				2.03 (95% CI: 1.48–2.79)	Tracheas, bronchus and lung, males		
				1.45 (95% CI: 1.32–1.58)	Natural causes, females		
				1.66 (95% CI: 0.93–2.95)	Tracheas, bronchus and lung, females		
				* Adjusted for age, calendar period, socio economic level, occupation in the ceramic industry, smoking sales and radon exposure			
Steinmaus C.M. et al., 2014 [35]	Non-occupational: arsenic drinking water	Never smokers Ever smokers Smoker > 10 cigs/day	OR	Unadjusted			Not reported
				1.00	[<10 μg/L]		
				1.46 (90% CI: 0.88–2.43)	[10–59.9 μg/L]		
				1.90 (90% CI: 1.16–3.13)	[>59.9 μg/L]		
				Adjusted *			
				1.00	[<10 μg/L]		
				1.43 (90% CI: 0.82–2.52)	[10–59.9 μg/L]		
				2.01 (90% CI: 1.14–3.52)	[>59.9 μg/L]		
				* Adjusted for age, sex and smoking behavior			
Steinmaus C.M. et al., 2013 [36]	Non-occupational: arsenic drinking water	Never smoker Ever smoker	OR	Adjusted for smoking			Synergistic
				1.00	[<11 μg/L]		
				1.27 (95% CI: 0.81–1.98)	[11–90 μg/L]		
				2.00 (95% CI: 1.24–3.24)	[91–335 μg/L]		
				4.32 (95% CI: 2.60–7.17)	[>335 μg/L]		
Hsu L. et al., 2013 [31]	Non-occupational: arsenic drinking water	Non-smokers Smokers	HR	Non-smokers *			Not reported
				1.00	Group 1 (no arsenical skin lesions)		
				0.24 (95% CI: 0.03–1.79)	Group 2 (hyperpigmentation only)		
				0.58 (95% CI: 0.08–4.26)	Group 3 (hyperkeratosis with or without hyperpigmentation)		
				3.24 (95% CI: 1.54–6.80)	Group 4 (skin cancer without hyperkeratosis)		
				2.22 (95% CI: 0.76–6.48)	Group 5 (skin cancer and hyperkeratosis)		
				Smokers *			
				2.20 (95% CI: 1.05–4.63)	Group 1 (no arsenical skin lesions)		
				1.69 (95% CI: 0.46–6.20)	Group 2 (hyperpigmentation only)		
				12.34 (95% CI: 4.79–31.75)	Group 3 (hyperkeratosis with or without hyperpigmentation)		
				12.04 (95% CI: 5.12–28.31)	Group 4 (skin cancer without hyperkeratosis)		
				23.54 (95% CI: 9.39–59.00)	Group 5 (skin cancer and hyperkeratosis)		
				* Hazard ratios were adjusted for age, sex, body mass index, educational level and cumulative arsenic exposure			
Ferreccio C. et al., 2013 [37]	Non-occupational: arsenic drinking water	Never smokers Ever smoker Heavier smokers (smoking > 10 cigs/day)	OR	Never smoker *			Greater than additive
				1.00	[0–34 μg/L]		
				0.87 (95% CI: 0.42–1.81)	[35–260 μg/L]		
				1.67 (95% CI: 0.78–3.56)	[>260 μg/L]		
				Smoked > 10 cigs/day *			
				4.36 (95% CI: 2.12–8.99)	[0–34 μg/L]		
				6.94 (95% CI: 3.48–13.83)	[35–260 μg/L]		
				20.80 (95% CI: 9.03–47.91)	[>260 μg/L]		
				* Adjusted for age, sex, socioeconomic status, and second-hand tobacco smoke exposure.			
Dauphiné D.C. et al., 2013 [38]	Non-occupational: arsenic drinking water	Never smoker Ever smoker	OR	All subjects			Not reported
				1.00	[≤10 μg/L]		
				0.84 (95% CI:0.40–1.79)	[11–84 μg/L]		
				1.39 (95% CI: 0.55–3.53)	[>85 µg/L]		
				Smokers			
				1.00	[≤10 μg/L]		
				0.66 (95% CI:0.30–1.44)	[11–84 μg/L]		
				1.61 (95% CI: 0.59–4.38)	[>85 µg/L]		
Chen C. e al., 2010 [32]	Non-occupational: arsenic drinking water	Never smokers Current smokers (<25 cigs/day) Current smokers (≥25 cigs/day)	RR	Never smokers			Synergistic for squamous and small cell lung cancer
				1.00	[<10 µg/L]		
				1.22 (95% CI: 0.64–2.32)	[10–99.9 µg/L]		
				1.32 (95% CI: 0.64–2.74)	[≥100 µg/L]		
				Current smokers (<25 cigs/day)			
				2.14 (95% CI: 0.79–5.79)	[<10 µg/L]		
				1.52 (95% CI: 0.56–4.15)	[10–99.9 µg/L]		
				5.30 (95% CI: 2.19–12.8)	[≥100 µg/L]		
				Current smokers (≥25 cigs/day)			
				4.08 (95% CI: 1.83–9.10)	[<10 µg/L]		
				4.19 (95% CI: 1.92–9.14)	[10–99.9 µg/L]		
				8.17 (95% CI: 3.74–17.9)	[≥100 µg/L]		
Paul S. et al., 2013 [41]	Non-occupational: arsenic drinking water	2005–2006 63 M, unexposed 34 W, unexposed 57 M, exposed 31 W, exposed 2010–2011 69 M, unexposed 39 W, unexposed 53 M, exposed 33 W, exposed	Lung cancer death (number)	2010–2011 *			Not reported
				1 individual among the unexposed died due to natural cause			
				3 males, exposed			
				0 female, exposed			
				* tobacco consumption were matched between unexposed and exposed subjects			
Marano K.M et al., 2012 [43]	Occupational and non-occupational	Cigarette smokers Smokeless tobacco (SLT) consumers Non-consumers of tobacco	Geometric mean of urine arsenic (μg/g creatinine)	39.1 for lung cancer Vs			Not reported
				7.98 (95% CI: 7.08–9.00)	Cigarette smokers *		
				6.14 (95% CI: 4.86–7.74)	SLT consumers *		
				9.56 (95% CI: 8.92–10.27)	Non-consumers of tobacco *		
				* without lung cancer			
Wadhwa S.K. et al., 2011 [39]	Non-occupational: water, food and fish; locally made cigarette	100% smokers	OR	Arsenic concentrations 3–15 fold higher than the permissible level (< 10 µg/L) 3.05 (95% CI: 1.26–7.36) (for lung cancer mortality among exposed lung cancer patients as compared to non-exposed lung cancer patients)			Synergistic
Olsson A.C. et al., 2011 [33]	Occupational: different kinds of industries, e.g., wood work and painting	Never smoker Former smoker Current smoker	OR	Arsenic < 50% TLV (2011) = low; 50–150% TLV (2011) = medium; >150% TLV (2011) = high Adjusted for smoking (tobacco pack years) Men: 1.92 (95% CI: 1.15–3.20) Women: 1.05 (95% CI: 0.11–9.89)			Not reported
’t Mannetje A. et al., 2014 [34]	Occupational: agriculture, miners and different kinds of industries	Never smokers Ex-smokers Ever smokers	OR	Arsenic concentration values not reported (low, medium and high exposure). Adjusted * 1.65 (95% CI: 1.05–2.58) * adjusted for age, center, sex, tobacco consumption and for other occupational exposures including metals			Not reported
Melak D. et al., 2014 [40]	Non-occupational: arsenic drinking water	Never smokers Smokers	OR	Water arsenic < 200 µg/L			Not reported
				Crude	Adjusted *		
				1.00	1.00	%MMA < 12.5%	
				2.65 (95% CI: 1.18–5.94)	2.48 (95% CI: 1.08–5.68)	%MMA ≥ 12.5%	
				Water arsenic ≥ 200 µg/L			
				Crude	Adjusted *		
				3.05 (95% CI: 1.56–5.95)	3.16 (95% CI: 1.59–6.32)	%MMA < 12.5%	
				6.94 (95% CI:3.39–14.22)	6.81 (95% CI:3.24–14.31)	%MMA ≥ 12.5%	
				* Odds ratios adjusted for age, gender and smoking			
				%MMA = % monomethylarsenic in urine; the cut-off value of 12.5% divided the upper from the two lower tertiles			

## Data Availability

Not available.

## Supplementary Materials

The following supporting information can be downloaded at: https://www.mdpi.com/article/10.3390/ijerph20054167/s1, Table S1: NOS values for the included studies. References [29,30,31,32,33,34,35,36,37,38,39,40,41,42,43,44] are cited in the Supplementary Materials.
