# Supplementary material for: Interaction between Occupational and Non-Occupational Arsenic Exposure and Tobacco Smoke on Lung Cancerogenesis: A Systematic Review
Source: Int J Environ Res Public Health. 2023 Feb 25;20(5):4167. doi: 10.3390/ijerph20054167 (PMC10001869; doi:10.3390/ijerph20054167)
Supplement: Supplementary file 1 [file ijerph-20-04167-s001.zip › ijerph-2209724-supplementary.pdf]

Supplementary Materials

Table S1: NOS values for the included studies.

Each author's study can be awarded a minimum of one star (*) and a maximum of four stars (****) for each numbered item, as reported in the Newcastle–Ottawa Assessment Scale (NOS) [27].

Su Z. [30]

COHORT STUDIES	Star	Total stars/group	Value
**SELECTION**		****	4
1) Representativeness of the Exposed Cohort	*		
2) Selection of the Non-Exposed Cohort	*		
3) Ascertainment of Exposure	*		
4) Demonstration That Outcome of Interest Was Not Present at Start of Study	*		
**COMPARABILITY**		**	2
1) Comparability of Cohorts on the Basis of the Design or Analysis	**		
**OUTCOME**		***	3
1) Assessment of Outcome	*		
2) Was Follow-Up Long Enough for Outcomes to Occur	*		
3) Adequacy of Follow Up of Cohorts	*		
**TOTAL STARS/ VALUES**		**nine**	**9**

Fan Y. [44]

COHORT STUDIES	Star	Total stars/group	Value
**SELECTION**		**	2
1) Representativeness of the Exposed Cohort	*		
2) Selection of the Non-Exposed Cohort			
3) Ascertainment of Exposure	*		
4) Demonstration That Outcome of Interest Was Not Present at Start of Study			
**COMPARABILITY**		*	1

1) Comparability of Cohorts on the Basis of the Design or Analysis	*		
**OUTCOME**		**	2
1) Assessment of Outcome<br>2) Was Follow-Up Long Enough for Outcomes to Occur<br>3) Adequacy of Follow Up of Cohorts	*<br>*		
**TOTAL STARS/ VALUES**		**five**	**5**

Steinmaus C.M. [29]

CASE CONTROL	Star	Total stars/group	Value
**SELECTION**		***	3
1) Is the Case Definition Adequate?<br>2) Representativeness of the Cases<br>3) Selection of Controls<br>4) Definition of Controls	*<br>*<br>*		
**COMPARABILITY**		*	1
1) Comparability of Cases and Controls on the Basis of the Design or Analysis	*		
**EXPOSURE**			2
1) Ascertainment of Exposure<br>2) Same method of ascertainment for cases and controls<br>3) Non-Response Rate	*<br>*		
**TOTAL STARS/ VALUES**		**six**	**6**

D'Ippoliti D. [42]

COHORT STUDIES	Star	Total stars/group	Value
**SELECTION**		*	1
1) Representativeness of the Exposed Cohort<br>2) Selection of the Non-Exposed Cohort<br>3) Ascertainment of Exposure<br>4) Demonstration That Outcome of Interest Was Not Present at Start of Study	*		

**COMPARABILITY**			
1) Comparability of Cohorts on the Basis of the Design or Analysis			
**OUTCOME**		***	3
1) Assessment of Outcome<br>2) Was Follow-Up Long Enough for Outcomes to Occur<br>3) Adequacy of Follow Up of Cohorts	*<br>*<br>*		
**TOTAL STARS/ VALUES**		**four**	**4**

Steinmaus C.M. [35]

**CASE CONTROL**	**Star**	**Total stars/group**	**Value**
**SELECTION**		****	4
1) Is the Case Definition Adequate?<br>2) Representativeness of the Cases<br>3) Selection of Controls<br>4) Definition of Controls	*<br>*<br>*<br>*		
**COMPARABILITY**		**	2
1) Comparability of Cases and Controls on the Basis of the Design or Analysis	**		
**EXPOSURE**		**	2
1) Ascertainment of Exposure<br>2) Same method of ascertainment for cases and controls<br>3) Non-Response Rate	*<br>*		
**TOTAL STARS/ VALUES**		**eight**	**8**

Steinmaus C.M. [36]

**CASE CONTROL**	**Star**	**Total stars/group**	**Value**
**SELECTION**		****	4
1) Is the Case Definition Adequate?<br>2) Representativeness of the Cases<br>3) Selection of Controls<br>4) Definition of Controls	*<br>*<br>*<br>*		
**COMPARABILITY**		**	2

1) Comparability of Cases and Controls on the Basis of the Design or Analysis	**		
**EXPOSURE**		**	2
1) Ascertainment of Exposure<br>2) Same method of ascertainment for cases and controls<br>3) Non-Response Rate	*<br>*		
**TOTAL STARS/ VALUES**		**eight**	**8**

Hsu L. [31]

**COHORT STUDIES**	**Star**	**Total stars/group**	**Value**
**SELECTION**		***	3
1) Representativeness of the Exposed Cohort<br>2) Selection of the Non-Exposed Cohort<br>3) Ascertainment of Exposure<br>4) Demonstration That Outcome of Interest Was Not Present at Start of Study	*<br>*<br>*		
**COMPARABILITY**		*	1
1) Comparability of Cohorts on the Basis of the Design or Analysis	*		
**OUTCOME**		*	1
1) Assessment of Outcome<br>2) Was Follow-Up Long Enough for Outcomes to Occur<br>3) Adequacy of Follow Up of Cohorts	*		
**TOTAL STARS/ VALUES**		**five**	**5**

Ferreccio C. [37]

**CASE CONTROL**	**Star**	**Total stars/group**	**Value**
**SELECTION**		***	3
1) Is the Case Definition Adequate?<br>2) Representativeness of the Cases<br>3) Selection of Controls<br>4) Definition of Controls	*<br>*<br>*		

**COMPARABILITY**		*	1
1) Comparability of Cases and Controls on the Basis of the Design or Analysis	*		
**EXPOSURE**		**	2
1) Ascertainment of Exposure<br>2) Same method of ascertainment for cases and controls<br>3) Non-Response Rate	*<br>*		
**TOTAL STARS/ VALUES**		**six**	**6**

Dauphiné D.C. [38]

**CASE CONTROL**	**Star**	**Total stars/group**	**Value**
**SELECTION**		****	4
1) Is the Case Definition Adequate?<br>2) Representativeness of the Cases<br>3) Selection of Controls<br>4) Definition of Controls	*<br>*<br>*<br>*		
**COMPARABILITY**		**	2
1) Comparability of Cases and Controls on the Basis of the Design or Analysis	**		
**EXPOSURE**		**	2
1) Ascertainment of Exposure<br>2) Same method of ascertainment for cases and controls<br>3) Non-Response Rate	*<br>*		
**TOTAL STARS/ VALUES**		**eight**	**8**

Chen C. [32]

**COHORT STUDIES**	**Star**	**Total stars/group**	**Value**
**SELECTION**		****	4
1) Representativeness of the Exposed Cohort<br>2) Selection of the Non-Exposed Cohort<br>3) Ascertainment of Exposure<br>4) Demonstration That Outcome of Interest Was Not Present at Start of Study	*<br>*<br>*<br>*		

**COMPARABILITY**		*	1
1) Comparability of Cohorts on the Basis of the Design or Analysis	*		
**OUTCOME**		**	2
1) Assessment of Outcome<br>2) Was Follow-Up Long Enough for Outcomes to Occur<br>3) Adequacy of Follow Up of Cohorts	*<br>*		
**TOTAL STARS/ VALUES**		**seven**	**7**

Paul S. [41]

**COHORT STUDIES**	**Star**	**Total stars/group**	**Value**
**SELECTION**		***	3
1) Representativeness of the Exposed Cohort<br>2) Selection of the Non-Exposed Cohort<br>3) Ascertainment of Exposure<br>4) Demonstration That Outcome of Interest Was Not Present at Start of Study	*<br>*<br>*		
**COMPARABILITY**			
1) Comparability of Cohorts on the Basis of the Design or Analysis			
**OUTCOME**		*	1
1) Assessment of Outcome<br>2) Was Follow-Up Long Enough for Outcomes to Occur<br>3) Adequacy of Follow Up of Cohorts	*		
**TOTAL STARS/ VALUES**		**four**	**4**

Marano K.M [43]

**COHORT STUDIES**	**Star**	**Total stars/group**	**Value**
**SELECTION**		*	1
1) Representativeness of the Exposed Cohort	*		

2) Selection of the Non-Exposed Cohort<br>3) Ascertainment of Exposure<br>4) Demonstration That Outcome of Interest Was Not Present at Start of Study			
**COMPARABILITY**			
1) Comparability of Cohorts on the Basis of the Design or Analysis			
**OUTCOME**		*	1
1) Assessment of Outcome<br>2) Was Follow-Up Long Enough for Outcomes to Occur<br>3) Adequacy of Follow Up of Cohorts	*		
**TOTAL STARS/ VALUES**		**two**	**2**

Wadhwa S.K. [39]

**CASE CONTROL**	**Star**	**Total stars/group**	**Value**
**SELECTION**		**	2
1) Is the Case Definition Adequate?<br>2) Representativeness of the Cases<br>3) Selection of Controls<br>4) Definition of Controls	 <br>*<br>*		
**COMPARABILITY**			
1) Comparability of Cases and Controls on the Basis of the Design or Analysis			
**EXPOSURE**		**	2
1) Ascertainment of Exposure<br>2) Same method of ascertainment for cases and controls<br>3) Non-Response Rate	*<br>* 		
**TOTAL STARS/ VALUES**		**four**	**4**

Olsson A.C. [33]

**CASE CONTROL**	**Star**	**Total stars/group**	**Value**
**SELECTION**		***	3
1) Is the Case Definition Adequate?	*		

2) Representativeness of the Cases<br>3) Selection of Controls<br>4) Definition of Controls	*<br>*		
**COMPARABILITY**		**	2
1) Comparability of Cases and Controls on the Basis of the Design or Analysis	**		
**EXPOSURE**		**	2
1) Ascertainment of Exposure<br>2) Same method of ascertainment for cases and controls<br>3) Non-Response Rate	*<br>*		
**TOTAL STARS/ VALUES**		**seven**	**7**

’t Mannetje A. [34]

**CASE CONTROL**	**Star**	**Total stars/group**	**Value**
**SELECTION**		***	3
1) Is the Case Definition Adequate?<br>2) Representativeness of the Cases<br>3) Selection of Controls<br>4) Definition of Controls	 *<br>*<br>*		
**COMPARABILITY**		**	2
1) Comparability of Cases and Controls on the Basis of the Design or Analysis	**		
**EXPOSURE**		**	2
1) Ascertainment of Exposure<br>2) Same method of ascertainment for cases and controls<br>3) Non-Response Rate	*<br>*		
**TOTAL STARS/ VALUES**		**seven**	**7**

Melak D. [40]

**CASE CONTROL**	**Star**	**Total stars/group**	**Value**
**SELECTION**		****	4
1) Is the Case Definition Adequate?<br>2) Representativeness of the Cases<br>3) Selection of Controls	*<br>*<br>*		

4) Definition of Controls			
**COMPARABILITY**		**	2
1) Comparability of Cases and Controls on the Basis of the Design or Analysis	**		
**EXPOSURE**		**	2
1) Ascertainment of Exposure<br>2) Same method of ascertainment for cases and controls<br>3) Non-Response Rate	*<br>*		
**TOTAL STARS/ VALUES**		**eight**	**8**
